# Professional Identity Formation in medical education and training – a discursive determination of the term in German-speaking contexts

**DOI:** 10.3205/zma001829

**Published:** 2026-03-23

**Authors:** Kristina Schick, Katja Kuehlmeyer, Barbara Jömann, Moritz Schumm, Sonja Mathes, Angelika Homberg

**Affiliations:** 1 Dresden University of Technology, Medical Faculty and University Hospital Carl Gustav Carus, Institute of Medical Education, Dresden, Germany; 2 Ludwig-Maximilians-Universität Munich, Institute of Ethics, History and Theory of Medicine, Munich, Germany; 3 Ruhr University Bochum, Medical Faculty, Institute of General Practice and Family Medicine, Bochum, Germany; 4 Technical University of Munich, Department Clinical Medicine and Health, TUM Medical Education Center, Munich, Germany; 5 Technical University of Munich, TUM School of Medicine and Health, Department of Dermatology and Allergology, Munich, Germany; 6 Heidelberg University, Medical Faculty Mannheim, Department of Medical Education Research, Division for Study and Teaching Development, Mannheim, Germany

**Keywords:** Professional Identity Formation, Professional Identity Development, Delphi technique, conceptual understanding, educational research, curriculum

## Abstract

**Objective:**

The concepts of Professional Identity Formation (PIF)/Professional Identity Development (PID) evolved in English-speaking countries with varying emphases. They serve as a starting point for the design of medical education, further education, and continuing training. The aim of this project was to obtain a discursive clarification of the conceptual understanding of PIF/PID in medical education research and curriculum development in German-speaking countries.

**Method:**

A shared understanding of the concept was developed using a structured discourse process (two workshops, an online survey, and a group Delphi). Individuals with different disciplinary backgrounds took part in the discourse. This was part of their work in a working group of the German-speaking Society for Medical Education (Gesellschaft für Medizinische Ausbildung, GMA). The analyses of the survey and the group Delphi were conducted by applying descriptive statistics and an argument-based analysis.

**Results:**

The group agreed upon the following understanding of the concept: “The professional identity development (Professionelle Identitätsentwicklung, PID) of physicians is an ongoing process in all phases of professional education and work. A professional identity develops in the interaction between person and environment. The process takes place both consciously and unconsciously. It is subject to internal and external influences that can be addressed and shaped in medical education, further education, and continuing training. In this process, individuals should acquire knowledge, skills and, in particular, develop a (self-)reflective attitude in the context of the norms and values of their profession.”

**Conclusion:**

This conceptual understanding of PID, which is compatible with medical education, further education, and continuing training, as well as with teaching and learning research, highlights the processual and influenceable nature of PID. Three follow-up questions arose from the discussions: (i) To what extent is PID a malleable process?, (ii) To what extent does PID pursue a normative claim?, and (iii) What is the relationship between PID and competence?

## Zusammenfassung

**Zielsetzung:**

Die Konzepte der „Professional Identity Formation“ bzw. der „Professional Identity Development“ entwickelten sich im angelsächsischen Raum mit unterschiedlichen Schwerpunktsetzungen. Sie dienen als Ausgangspunkt für die Gestaltung der ärztlichen Aus-, Weiter- und Fortbildung. In diesem Projekt wurde die diskursive Klärung des Begriffsverständnisses für den deutschsprachigen Raum im Forschungs- und Praxisfeld der Medizindidaktik angestrebt.

**Methode:**

In einem Diskursverfahren (zwei Workshops, Online-Befragung und Gruppendelphi) wurde ein gemeinsames Begriffsverständnis erarbeitet. Am Diskurs nahmen Personen mit unterschiedlichen disziplinären Hintergründen im Rahmen ihrer Mitarbeit in einer Arbeitsgruppe der Gesellschaft für Medizinische Ausbildung (GMA) teil. Die Auswertungen der Befragung und des Gruppendelphis erfolgten anhand deskriptiver Statistik und einer Analyse der Argumente.

**Ergebnisse:**

Folgendes Begriffsverständnis wurde konsentiert: „Die professionelle Identitätsentwicklung (PIE) von Ärzt:innen ist ein fortdauernder Prozess in allen Phasen der berufsbezogenen Ausbildung und Tätigkeit. Die professionelle Identität entwickelt sich in der Interaktion zwischen Person und Umwelt. Der Prozess verläuft sowohl bewusst als auch unbewusst. Er unterliegt inneren und äußeren Einflüssen, die in der ärztlichen Aus-, Weiter- und Fortbildung aufgegriffen und gestaltet werden können. In diesem Prozess sollen Personen im Kontext der Normen und Werte ihrer Profession sich Wissen aneignen, Fertigkeiten erwerben und insbesondere eine (selbst-)reflexive Haltung entwickeln.“

**Schlussfolgerung:**

Das an die Aus-, Weiter- und Fortbildung sowie die Lehr-Lernforschung anschlussfähige Begriffsverständnis betont die prozesshafte und beeinflussbare Entwicklung professioneller Identität. Durch die Diskussionen ergaben sich drei Anschlussfragen: (i) Inwieweit ist PIE ein gestaltbarer Prozess? (ii) Inwiefern wird mit PIE ein normativer Anspruch verfolgt? (iii) Wie gestaltet sich das Verhältnis von PIE und Kompetenz?

## 1. Introduction

The question of how medical students and physicians (in training) can be supported in developing their (future) professional roles has been increasingly discussed in recent years under the concepts of “Professional Identity Formation” (PIF) and “Professional Identity Development” (PID) [1]. The terms were introduced in medical education research in the early 2010s, particularly in English-speaking countries, including the USA and Canada [2]. In light of complex social demands placed on physicians, the Carnegie Foundation called for PIF/PID to be integrated into medical education. Following this call, the number of scientific publications in this area increased significantly [3].

One of the first definitions of PIF/PID is provided by Merton ([4], p.7), who states that the task of medical education, further education, and continuing training should be to convey a medical, intra-professional culture capable of equipping (prospective) physicians with a professional identity that allows them “to think, act, and feel like a physician”. Sternszus et al. [5] build on this definition, and specify PIF as a process of internalizing professional characteristics, values, and norms that shape a physician’s self-image. Other authors also emphasize the assumption of the medical role and the associated affiliation to the medical profession [6], [7]. The first systematic reviews, including the scoping review by Sarraf-Yazdi et al. [8], provide complex explanations. Sarraf-Yazdi et al. [8] developed a model in which PIF occurs as a continuum resulting from the interplay of (inter-)individual and social factors. According to this model, a sense of belonging to the profession emerges when the self-perceived, aspired, and actual identities are in alignment [9]. Holden et al. describe PIF as a “transformative journey” in which acquired competencies are integrated into one’s core values and in which professional and personal growth are interconnected ([10], p.762). To capture the complexity of this process, they suggest a framework consisting of ten key factors and six domains [10]. A systematic review of the literature by Schumm et al. [11] provides a comprehensive overview of current definitions and their emphases.

Given the diverse international research contributions, a consensual understanding of the terms and concepts associated with PIF/PID is desirable to enable German-speaking countries to leverage the potential of PIF/PID for teaching, curriculum development, and research [8]. The term “conceptual understanding” refers here to heterogeneous statements that, on the one hand, facilitate understanding of a word’s meaning while, on the other hand, provide insights which can facilitate project planning. In recent years, there has been an increase in relevant, but very different teaching concepts for implementing PIF/PID in German-speaking countries. For example, one aim of narrative medicine is to facilitate the discussion of inter-individual horizons of meaning between physicians and patients [12]. So far, the focus of training has been on the development of individual teaching and support programs, as well as on longitudinal curricula in medical education [13], [14], [15], [16], [17]. Thus, a comprehensive understanding of PIF/PID for medical education, further education, and continuing training, as well as for educational research, is still lacking in German-speaking countries. 

The aim of this project is to develop a discursive determination of the terms PIF/PID for German-speaking countries. This can then form the basis for developing teaching and support programs for PIF/PID in medical education, further education, and continuing training, as well as providing a conceptual basis for educational research on PIF/PID. The project was conducted as part of the “Professional Identity Formation” working group of the German-speaking Society for Medical Education (Gesellschaft für Medizinische Ausbildung, GMA), established in 2021. In this article, we outline the discursive process used to develop a shared conceptual understanding, and we present the consented result.

## 2. Method

### 2.1. Institutional context

Only members of the GMA working group “Professional Identity Formation” participated in the discursive determination of the term. This group comprises individuals with backgrounds in medicine, social sciences, and humanities in medical education contexts. It provides a platform for interested individuals from German-speaking countries to discuss the concept of PIF/PID, along with the associated content-related and methodological challenges. Members are usually involved in teaching projects or curriculum development at medical schools. The physicians can also contribute experiences from their own PIF/PID process. Within the GMA working group, the subgroup “Definition & Theoretical Foundations” was formed to develop a shared understanding of the concept of PIF/PID. As part of the subgroup, the authors of this article have established a writing group. All members of the entire working group were able to participate in the discursive process of determining the term.

### 2.2. Procedure for determining the term

The determination of the term was conducted through a multimodal discourse in three phases:

1. Development of an initial understanding of the concept: Face-to-face Workshop I (January 2023)
2. Discussion of the first understanding of the concept:
3.
  1. An asynchronous online survey with a structured semi-open questionnaire (May to August 2023)
  2. Face-to-face Workshop II (January 2024)
4. Consensus building on the understanding of the concept: Online group Delphi in two rounds, with a concluding online workshop (May 2024 to October 2024)

Throughout the entire process, all opinions and attitudes could be expressed in an open, argumentative exchange, and all participants were entitled to the same rights. An intention to publish was communicated since the second phase of the process. The first two phases were open to new participants from the working group; however, during the third phase, participation in the first Delphi round was a prerequisite for participation in the remaining process. The three phases of the discourse are presented below. 

#### 2.2.1. Developing an initial understanding of the concept: Face-to-face workshop I 

As part of a workshop organized by the GMA working group, eight academic articles on PIF were circulated among the participants. The articles were compiled in advance based on suggestions from the working group members. The selection was made on the basis of concise and, as far as possible, diverse examples. This approach allowed the participants to gain an overview of the existing perspectives on the topic of PIF/PID. A synopsis was created in advance and also made available (see attachment 1 ). During the face-to-face workshop, the participants were divided into two groups. Each group was asked to analyze four definitions in terms of their structure, core content elements, and intentionality. The findings were clustered on a board, presented, and discussed. This discussion inspired an initial formulation of a shared conceptual understanding of PIF/PID. This was continuously discussed and gradually adapted until a first draft was produced, which the participants agreed on at the time. All working group members were then informed about the workshop by means of minutes and a progress report.

#### 2.2.2. Discussion of the understanding of the concept

##### 2.2.2.1. Asynchronous online survey with a structured, semi-open questionnaire 

The initial draft was further developed through an anonymous online survey using the Unipark survey tool [https://www.tivian.com]. All members of the PIF working group (n=46) were invited via email to participate in the survey. 

The individual sentences of the draft conceptual understanding were assessed on a 4-point Likert scale (1=“strongly disagree” to 4=“agree”). In addition, the assessments could be explained and alternative wording could be suggested. 

The analysis was conducted descriptively and statistically by calculating the median (Md) and the interquartile range (IQR). The free-text responses were reviewed, summarized, and structured by three of the authors (KS, SM, and AH) using the Argument-based QUalitative Analysis strategy (AQUA) (see attachment 2 ) [18].

##### 2.2.2.2. Face-to-face workshop II

The results of the online survey were discussed in a subsequent workshop. Prior to the workshop, participants received a written summary of the survey results and a detailed list of the free-text responses. At the beginning of the workshop, the key points of agreement and disagreement regarding the individual components of the conceptual understanding were summarized. The participants were subsequently divided into three groups. Group A worked on the first two sentences of the suggested conceptual understanding of PIF, group B worked on the middle sentence, and group C worked on the last two sentences. All three groups used the following work assignments:

1. Formulate three alternative sentences considering the survey results.
2. Name the most controversial point of discussion.

Ninety minutes were set aside for the group work. The results were recorded, presented, and discussed.

#### 2.2.3. Reaching a consensus on the understanding of the concept: Online group Delphi

##### 2.2.3.1. The group Delphi method

During the consensus-building phase, we based our discourse on the group Delphi approach. The group Delphi method is a modified and mixed-methods version of the traditional Delphi method for expert discourses [19]. The application of the method involves the combination of a quantitative analysis of answers to a questionnaire and a qualitative analysis of a real-time discussion process. The group Delphi process aims at establishing consensus and identifying reasons for dissent, while exploring whether dissent can be resolved through communication [20]. 

A key feature of the group Delphi process is that it generates group responses rather than individual responses. These group responses should first be elicited in small groups and then discussed in the plenary session. The questionnaire for the group Delphi process is typically tailored to the specific project. The method allows participants to exchange views on different assessments [20].

##### 2.2.3.2. Participants

For the group Delphi process, it was intended to recruit 12 to 20 participants from the PIF working group. Participants should represent different backgrounds (discipline, work context, professional role, gender, and career stage). 

##### 2.2.3.3. Group Delphi process

**Figure 1:**
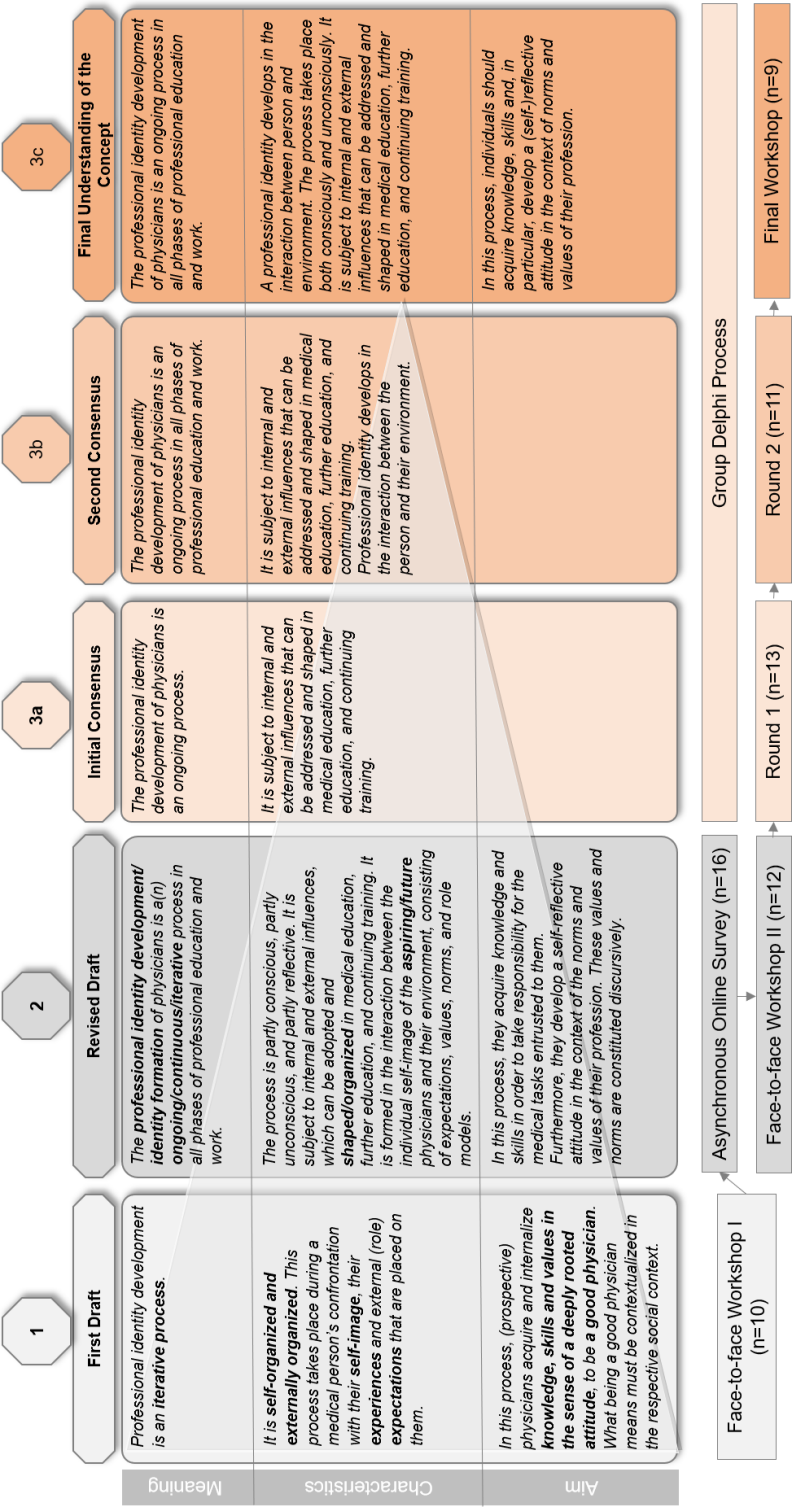
Development of the concept of Professional Identity Formation

The suggested modifications in Workshop II were the basis for the questionnaires used in the group Delphi process. The questionnaire was divided into four parts (A-D), and modified throughout the rounds of the group Delphi. Part A explained the subject of the concept (meaning), Part B contained aspects that describe the process of PIF/PID (characteristics), and Part C explained what PIF/PID should achieve (aims) (see figure 1 (Fig. 1)). The statements in parts A-C were evaluated on an eight-point rating scale (1=“no agreement” and 8=“full agreement”). 

Part D (designation of the term) dealt with the question of how the result should be labeled (e.g., definition, understanding of the concept, determination of the term). Due to the difficulties in arranging joint appointments, the process was divided into smaller sections and organized partly asynchronously. 

Each online meeting was structured as follows: After an introduction to the working process, participants were divided into small groups of three to four people to find group answers to the survey questions. Abstentions or two answers per item should only occur in exceptional cases. The small group discussions were facilitated and recorded by one or two members of the organizational team (KS, KK, and AH). Following each online meeting, the participants received feedback on the results of the previous Delphi round.

##### 2.2.3.4. Analysis

Group responses to sections A to C were analyzed using descriptive statistics to determine measures of location and dispersion (median (Md), interquartile range (IQR). The consensus criterion was an IQR<2 (see [17]). At least average ratings (scale level≥5) were considered to indicate agreement. In cases of wide variation or lack of agreement, statements were discussed within the organizational team and modified for the next round. Statements were deleted if there was consensus on their rejection.

We used the AQUA strategy [18] to analyze the minutes of the meetings. First, AH and KK each analyzed the statements independently, and then they discussed their individual results and agreed on the joint results. 

##### 2.2.3.5. Ethical aspects

As all participants took part in the discourse voluntarily, on their own responsibility within a working group and were able to withdraw at any time, their participation posed minimal risks or potential burdens. In the discourse, we emphasized equal opportunities, respect, and transparency. Personal data were only collected with the consent of the respective participants and were used solely for the purpose of organizing the discourse. As this is not a medical research project but a discourse of a working group, no ethics approval by a research ethics committee was obtained. 

## 3. Results

### 3.1. Participation in the communication process

The participants came from a variety of disciplinary backgrounds (see table 1 (Tab. 1)). 

#### 3.1.1. Developing an initial understanding of the term: Face-to face workshop I

The discussion of the synopsis in Workshop I revealed three key aspects that distinguished the respective definitions:

1. a description of PIF/PID as a process or as a trait;
2. features that characterize PIF/PID; and
3. the degree of concretization and the use of empty phrases.

*Regarding 1:* On the one hand, PIF/PID has been described as a kind of stable personal characteristic, in the sense of a desirable trait [19], regarding how a medical person should “think, act, and feel” [4], [21], [22]. On the other hand, PIF/PID has also been described as a process or development towards a goal, in the sense of a desirable state, which can change over time and in different situations [1], [2], [7], [19]. The participants agreed to describe PIF/PID as a process that can be controlled both internally and externally. 

*Regarding 2:* Previous conceptual understandings included characterizations of PIF/PID that relate, for example, to norms, values, attitudes, knowledge, and skills [5], [6], [10]. 

*Regarding 3:* The participants found the fact that no concrete suggestions were made on how to deal with these aspects particularly noteworthy [5], [10] but instead a reference to overarching elements of medical education, further education, and continuing training, was made, such as ability to reflect, having role models, and providing feedback [5], [6]. 

Based on these considerations, the participants developed a first draft of their conceptual understanding of PIF documented as an intermediate result (see figure 1 (Fig. 1), step 1).

### 3.2. Discussion of the understanding of the concept

#### 3.2.1. Asynchronous online survey with a structured, semi-open questionnaire

Sixteen individuals took part in the online survey (response rate 34.8%). The results show that despite average medium to high approval rates (Md≥2), criticism was frequently expressed and improvements suggested (see attachment 2 ).

- *Sentence 1: *There was a high level of agreement that PIF/PID constitutes a process. However, there were very different interpretations of whether the process is “iterative”. In addition, the context in which the process is taking place was not mentioned.
- *Sentence 2:* Whether the process can be “self-organized and externally organized” was questioned. The fact that PIF/PID “takes place” suggests passivity, whereas an “organized” process conveys that PIF/PID can take place deliberately. Participants emphasized that PIF/PID can also occur passively and unconsciously.
- *Sentence 3: *The statement that self and role expectations, as well as experiences, could influence PIF was perceived as too complex and not very meaningful. Here, it would be helpful to clarify which aspects could be influenced internally and which externally.
- *Sentence 4:* A comparison between PIF/PID and competence was made. Participants suggested to integrate the elements of knowledge, skills, and attitudes into the understanding of the concept.
- *Sentence 5: *When describing the aim of PIF/PID, a reference was made to the “good physician”. A lack of detail of social contextualization of a good physician was raised. The statement was considered imprecise and therefore arbitrary (see figure 1 (Fig. 1), steps 2–5).

#### 3.2.2. Face-to-face workshop II 

During Workshop II, participants discussed the results of the online survey and made reformulations. First, the focus was on the translation of PIF/PID into German, and the use of the meaning of the term “development” (“Entwicklung”) instead of “formation” (“Bildung”) was discussed. The process description “iterative” was perceived as mathematical and technical; in contrast, alternatives such as “ongoing” and “continuous” were discussed. It was considered that PIF/PID should be characterized not only as a “conscious” or “unconscious” process but also as a “reflective” process. The importance of the interplay between “internal attitude” and “external influences” was also emphasized. An overview of further discussion points can be found in attachment 2 . 

The organizers revised the result of the discussion with regard to linguistic coherence (see figure 1 (Fig. 1), step 2).

### 3.3. Reaching a consensus on the conceptual understanding: Online group Delphi

In the first round of the modified group Delphi, a distinction was drawn between the terms “identity formation” and “identity development.” The group then reached consensus on the translation of PIF/PID as “Professionelle Identitätsentwicklung” (which corresponds more with the English term professional identity development, PID). It was argued that the term “formation” refers more to external influences, appears static or passive, and could suggest a proximity to medical education. This implies that a professional identity is developed within a limited and narrow education program, which does not acknowledge that PID also takes place alongside professional work. From now on, the term PID is used to describe the concept in question. We deviate from this when we refer to other works that explicitly use the term “Professional Identity Formation (PIF)”. 

There was a consensus that PID is an ongoing process. Characterizing PID as “continuing” has no added value, while the term “iterative” implies a process that repeats itself in loops, which does not have to be the case. A description of PID as “ongoing” was therefore preferred, as this emphasizes an open-ended development process. However, there were concerns that this could incorrectly imply a linear progression.

The participants agreed that PID can occur both consciously and unconsciously. However, the ability to reflect cannot simply be assumed, but must be developed and encouraged. In order to promote this ability, (future) physicians should be supported in making unconscious processes explicit and thus shapeable. 

Some participants criticized the reference to “internal and external influences,” arguing that external influences point to a political dimension and are sometimes not explicitly described (e.g., in the hidden curriculum). In addition, this aspect appeared to be dispensable, as it was directly linked to the aspect of “conscious and unconscious”. However, the participants did not provide a more precise definition of what they meant by “internal and external influences” (see attachment 3 ). When framing PID within the context of medical education, further education, and continuing training, it was argued that although this opens up a space for medical education, it also artificially narrows the scope of PID to formal education programs. PID can take place in all phases and areas of medical practice, but also beyond and without specific external intervention. The consented statements after the first round of the group Delphi are shown in figure 1 (Fig. 1), step 3a.

In the second round and in the concluding workshop, agreement was reached that the concept should include a normative claim. Furthermore, a programmatic mandate for medical schools to promote self-reflection skills could be derived from this claim. On the other hand, it was argued that PID would still be possible even without this mandate and that some form of identity could always be established at the end of the program. This raised the question of whether PID should also be used to achieve a specific normative goal. 

By rejecting the concept of competence, while at the same integrating references to the acquisition of knowledge and skills, different positions on the compatibility of competence orientation with PID became apparent. 

The result of the second round of the group Delphi is shown in figure 1 (Fig. 1), step 3b. An overview of these and additional discussion points from the individual sections of the group Delphi is presented in attachment 3 . The consented final result of the discourse is shown in figure 1 (Fig. 1), step 4.

## 4. Discussion

This article describes the cultural translation of the concept “Professional Identity Formation” into the German context using the term “Professionelle Identitätsentwicklung”. Compared to other discursive determinations of a term, one strength of this work is that it not only reports the results, but also makes the discourse transparent and explains the reasons for the respective wording. This work has the potential to guide the development of teaching programs and related initiatives, as well as informing the subject of research. It surpasses other determinations of the term (see attachment 1 ).

After the translation of the concept and its associated discourses into the German-speaking context, the following three elements require further discussion and clarification: The question

1. whether PID can even be understood as a process that can be shaped,
2. of the extent to which the determination of the term should also give rise to a normative claim, and
3. of the relationship between PID and competency.

All three aspects relate to socio-cultural differences in comparison between the Anglo-American and the German context regarding the understanding and use of terms. Significant differences between medical education, further education, and continuing training in Germany and the USA lie, on the one hand, in the prerequisites and timing of licensure (Germany: Licensure is attained after a successful final exam after the study program, USA: Licensure is attained only after one to two years of residency following university, [23], [24]). On the other hand, the financing of medical education varies greatly. In Germany, European students study for free, whereas students in the USA have to pay high tuition fees [25].

- *Regarding 1: *To emphasize the processual nature of the concept, the German term *“Professionelle Identitätsentwicklung*” was chosen. This translation corresponds more with the term “Professional Identity Development” [26], [27] and differs from the term “Professional Identity Formation” (In German: “Professionelle Identitätsbildung”) [2], [21], which is more established in English-speaking countries. “Identitätsbildung” could be associated with the German term ”Bildung”, which emphasizes more an institutionalized context. *“Professionelle Identitätsentwicklung”*, however, implies a process that is never fully complete [8], [26], [28] and therefore cannot be limited to medical education. This raises the question to what extent PID already occurs spontaneously and without the explicit support of educational programs, and whether every young professional develops a professional identity, or whether they already have one in some form and continues to develop it. In this context, the question arises as to what extent the success of PID can be questioned from the outside. One might assume that any identity that forms the basis of a professional activity as a physician can be considered sufficiently successful. However, the conceptual understanding of PID developed here includes reflection as a lever for a consciously shaping development and adapting it to requirements. Depending on which requirements are taken into consideration, (future) physicians could therefore decide to actively shape their identity development – for example, to be perceived as trustworthy physicians based on patient preferences [29] or to define their own objectives for occupying leadership positions based on reflection of gender differences at various career stages [30]. In medical education, this can also be addressed at an early stage, for example by discussing and reflecting on ethical and moral dilemmas in concrete, practical cases in order to develop courses of action and coping strategies for internal conflicts [15].
- *Regarding 2:* The proposed conceptual understanding has a normative character, as it calls for the involvement of external parties, such as medical educators. However, it lacks a normative orientation, in that there is no clear determination of the specific norms and values of the profession that are relevant to PID. The result of the group Delphi could therefore be criticized as being vague and arbitrary, just like the first draft after Workshop I, which referred to virtues (of the “good physician”). On the one hand, determining relevant norms and values for the medical profession would be important for PID, on the other hand, they are subject to constant change. Looking at the Hippocratic Oath, for example, it becomes clear that while some professional ethical norms, such as confidentiality and abstinence, are still valid today, others, such as the ban on performing surgery or the financial security of educators, are outdated. In addition, other issues, such as the ban on abortion and euthanasia, have been the subject of heated debate in German-speaking countries in recent decades and have partially been (juridically) liberalized [31]. Furthermore, current documents on professional ethics and law, such as the International Code of Medical Ethics or the Declaration of Geneva of the World Medical Association, as well as the Model Professional Code for Physicians Working in Germany by the German Medical Association, are regularly revised and adapted to social developments [32], [33], [34]. Nevertheless, particularly in view of historical developments such as the “destruction of life unworthy of living” [35], which guided the actions of some physicians during the Nazi era, norms and values that are decisive for the medical profession must not be left to arbitrariness. In summary, PID is taking place against the backdrop of the requirements resulting from the constantly changing, values and norms that are fundamentally humanistic in nature. Another normativity arises in considerations regarding the examination of PID, for which the conceptual understanding in this article is not yet sufficient. Without this aim of an education process, no normative assessment in the sense of successful or unsuccessful PID can take place. Sarraf-Yazdi et al. [36], for example, describe the goal of PIF/PID as achieving the greatest possible alignment between desired and existing identity [8], [36].
- *Regarding 3: *Given the breadth and diversity of medical practice, it is assumed that there is not just one identity within a “community of practice,” but rather a variety of identities due to the existence of multiple communities (“landscape of practice”) [37]. If a person can activate different identities depending on the situation, the question arises as to how the concept of competence can be distinguished from PID. In addition to “knowledge” and “skills”, “attitudes” also represent an important pillar of the concept of competence [38], [39], [40]. These aspects are also reflected in the reported shared conceptual understanding. To be perceived as competent, knowledge, skills, and attitudes must be able to be applied flexibly and successfully in a variety of problematic situations [40]. Performance (i.e., the observable implementation of competence) can vary depending on the situation or role an individual assumes. In the discourse on PID, the question arises as to how integration and simultaneous differentiation from the concept of competence can be achieved. There are certainly possibilities for integration: On the one hand, learning objectives can be identified in the National Competence Based Catalogue of Learning Objectives (Nationaler Kompetenzbasierter Lernzielkatalog Medizin, NKLM) that are relevant to PID (e.g., considering “professional values & norms” or reflecting on “personal needs & prerequisites”). In contrast, PID could also be understood as an overarching umbrella concept that provides a conceptual framework for specific medical role patterns (e.g., CanMEDS roles [41]).

Following this project, the applicability of the determination of the term in German-speaking countries should be examined. However, the discourse here refers exclusively on the medical profession, which is why specific characteristics of this professional group are likely to have influenced the results (such as the potential individual significance of coming from a family of physicians or the continuation of professional identity development in retirement). Transferability to other healthcare professions could, for example, be discussed in follow-up projects. Numerous examples stem from English-speaking countries where PID has also been considered in the context of nursing, physiotherapy, and pharmacy, for example [42], [43]. 

### 4.1. Limitations

The process of this discourse was not predetermined. It developed dynamically from the engagement of the members of the GMA PIF working group. This was already evident in the selection of definitions for the first workshop, where we had asked the working group members which definitions or articles they preferred to reference. Although we selected frequently cited articles, this process did not ensure that all relevant perspectives were available. Another critical point is that the participants were not specifically selected based on their expertise but were drawn exclusively from the PIF working group. However, it is particularly noteworthy that all participants demonstrated a high level of commitment and willingness to take part in this discourse despite the time constraints and demands of their jobs. This demonstrates the participants’ high level of engagement in expanding knowledge about PID and their strong intrinsic motivation. Since all participants belonged to the PIF working group, in which educators and researchers from German-speaking countries exchange views on the concept, the assumption can be made that a great variety of German-speaking experts have been involved in the process.

Although we made every effort during all phases of the discourse process to conduct a determination of the term based on the diverse opinions, attitudes, and backgrounds of the participants, it is possible that important perspectives may have been overlooked. No participants who worked in German-speaking countries, such as Switzerland or Austria, nor from the field of further education and continuing training could be included in the process. The heterogeneity of the group also made the consensus process challenging, and not only in terms of content. There were also barriers to finding suitable dates, meaning that the group Delphi rounds were largely asynchronously and tasks such as exchanging arguments between subgroups and reformulating proposals had to be taken over by the organization team. Due to social pressure to conform, participants may have felt compelled to align themselves with a position that seemed to be “acceptable to the majority”, particularly in the concluding workshop.

## 5. Conclusion

As a result of the multi-stage process reported in this paper, consensus was reached on the following determination of the term: “The professional identity development (Professionelle Identitätsentwicklung) of physicians is an ongoing process in all phases of professional education and work. A professional identity develops in the interaction between person and environment. The process takes place both consciously and unconsciously. It is subject to internal and external influences that can be addressed and shaped in medical education, further education, and continuing training. In this process, individuals should acquire knowledge, skills and, in particular, develop a (self-)reflective attitude in the context of norms and values of their profession.” 

PID has the potential to be an important point of reference in medical education, further education, and continuing training. The determination of the term presented here, provisional as it may be, can provide a starting point for specific or longitudinal teaching, support, and research projects in medical education. It can be used to design teaching methods for medical education, further education, and continuing training in such a way that PID is addressed. 

## Acknowledgements

We express our gratitude to all participants in the workshops, the survey, and group discussion. We are grateful to Juliane Walther for the collaboration during this project. We thank the speakers of the PIF working group, the editors of the special issue as well as the anonymous reviewers. 

## Authors’ ORCIDs

- Kristina Schick: [0000-0002-4819-4604]
- Katja Kuehlmeyer: [0000-0003-2839-8850]
- Barbara Jömann: [0009-0006-9852-4217]
- Moritz Schumm: [0009-0008-2663-7815]
- Sonja Mathes: [0000-0001-5770-762X]
- Angelika Homberg: [0000-0001-5585-1126]

## Competing interests

The authors declare that they have no competing interests. 

## Supplementary Material

Attachment 1: Phase 1 – Material used in workshop 1

Attachment 2: Phase 2

Attachment 3: Phase 3

## Figures and Tables

**Table 1:**
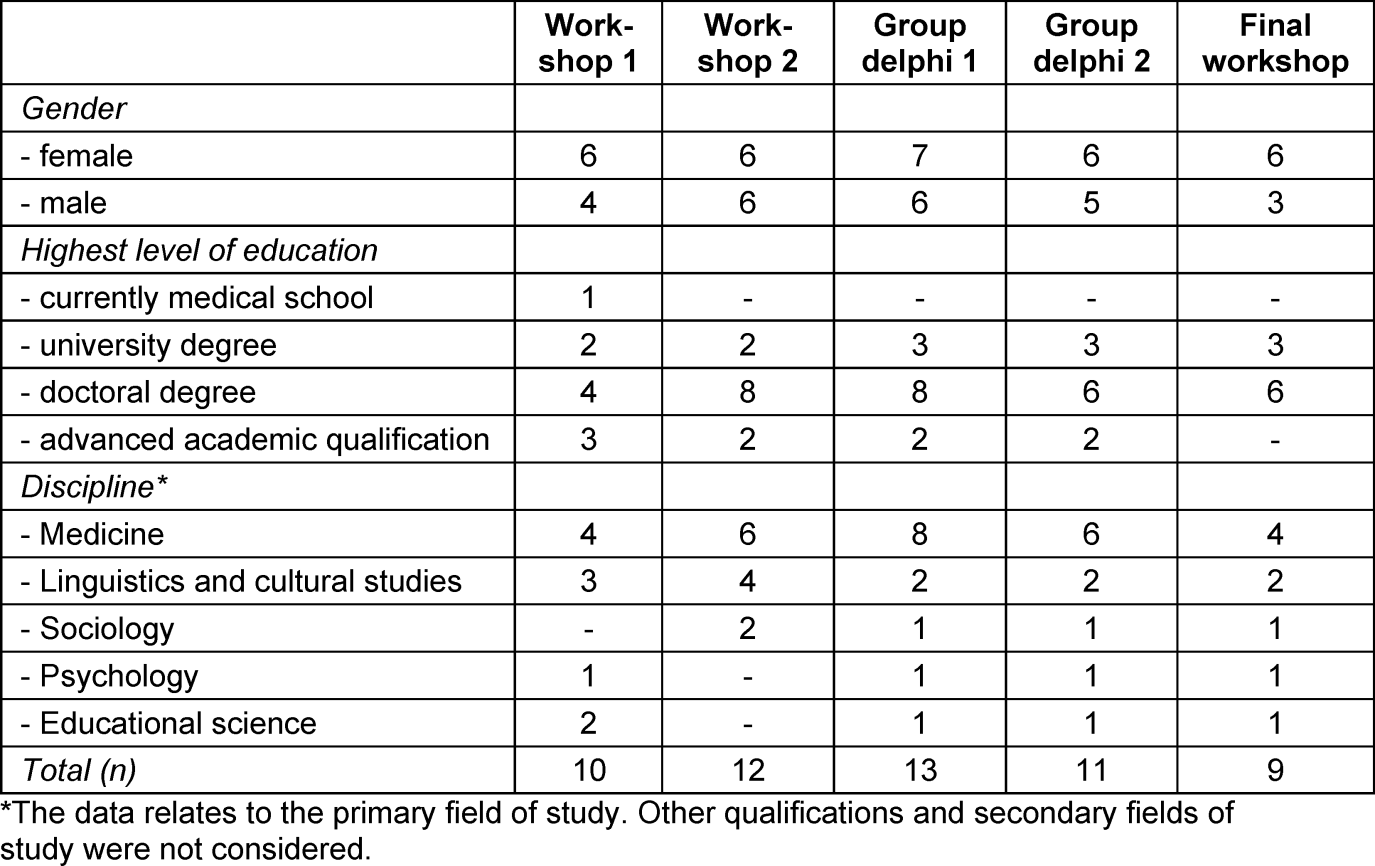
Participant demographics
