# Supplementary material for: Professional Identity Formation in medical education and training – a discursive determination of the term in German-speaking contexts
Source: GMS J Med Educ. 2026 Mar 23;43(3):Doc35. doi: 10.3205/zma001829 (PMC13054823; doi:10.3205/zma001829)
Supplement: Attachment 1: Phase 1 – Material used in workshop 1 [file JME-43-35-s-001.pdf]

Attachment 1: Phase 1 – Material used in workshop 1

Attachment 1 to Schick K, Kuehlmeyer K, Jömann B, Schumm M, Mathes S, Homberg A. *Professional Identity Formation in medical education and training – a discursive determination of the term in German-speaking contexts*. GMS J Med Educ. 2026;43(3):Doc35. DOI: 10.3205/zma001829

Attachment 1: Phase 1 – Materials used in workshop 1

Literature	Included Description of PIF
Forsythe GB. Identity development in professional education. Acad Med. 2005;80(10):S112-7. DOI: 10.1097/00001888-200510001-00029	“We want them to have the values of the profession; we want them to be ethical, to reason about the ethical implications of practice. And we want them to be doctors, or lawyers, or engineers, or teachers, or ministers, or nurses, or officers; we want them to have a sense of themselves as professionals.“ (S. S112)
Helsper W. Professionalität und Professionalisierung pädagogischen Handelns: Eine Einführung. [Professionalism & professionalization of educational practice: An introduction.] Stuttgart: UTB; 2021. Opladen, Toronto: Verlag Barbara Budrich.	„Professionalism is not only demonstrated in the success of professional client relationships and the successful resolution of crises, but also in situations and processes of failure or the failure of crisis management. Because professional action is not according to the rulebook and causal recipes, but is a complex social, meaning-structured interactive process that is susceptible to disruption, situational and case-specific and therefore fragile, it remains susceptible to failure and mistakes - such as the famous “malpractice” in medical practice or misdiagnosis in pedagogical practice. It is precisely then, however, that professionalism proves itself in the reflexive handling and (self-)critical collegial discussion of these sources of error and potential for error.“ [translated by the authors] (S. 56)
Holden MD, Buck E, Luk J, Ambriz F, Boisaubin EV, Clark MA, Mihalic AP, Sadler JZ, Sapire KJ, Spike JP, Vince A, Dalrymple JL. Professional identity formation: creating a longitudinal framework through TIME (Transformation in Medical Education). Acad Med. 2015;90(6):761-7. DOI: 10.1097/ACM.0000000000000719	„Professional Identity Formation is the transformative journey through which one integrates the knowledge, skills, values, and behaviors of a competent, humanistic physician with one’s own unique identity and core values. This continuous process fosters personal and professional growth through mentorship, self-reflection, and experiences that affirm the best practices, traditions, and ethics of the medical profession. The education of all medical students is founded on PIF.“ (S. 762)
Irby DM, Hamstra SJ. Parting the clouds: Three professionalism frameworks in medical education. Acad Med. 2016;91(12):1606-11. DOI: 10.1097/ACM.0000000000001190	“Professional identity formation focuses on both the individual and the group and, like the virtue-based framework, explores internal developmental processes of being and becoming versus doing. The dominant assumption in this framework is that professionalism involes becoming a good doctor by aspiring to attain a certain professional identity, as depicted by positive role models. This perspective goes beyond the virtue- and behavior-based frameworks by acknowledging the powerful social forces implicit in becoming part of a community of practice.” (S. 1609)
Jarvis-Selinger S, Pratt DD, Regehr G. Competency is not enough: integrating identity formation into the medical education discourse. Acad Med. 2012;87(9):1185-90. DOI: 10.1097/ACM.0b013e3182604968.	„Reflection on the relationship between social roles, professional identity, and individual competence specific to a particular community of practice is, therefore, the critical process linking social structures with individual behavior. Whereas society provides roles that are the basis of identity, the emerging self is the “active creator of social behavior.” Understanding the interplay between the social and personal aspects of identity formation allows a fuller appreciation of the complexity with which various overlapping physician roles emerge.“ (S. 1189f)
Sternszus R, Boudreau JD, Cruess RL, Cruess SR, MacDonald ME, Steinert Y. Clinical teachers‘ perception of their role in professional identity formation. Acad Med. 2020;95(10):1594-9. DOI: 10.1097/ACM.0000000000003369	„Professional identity formation is a developmental process whereby the characteristics, values, and norms of the profession are internalized as medical students and residents learn to think, act, and feel like physicians.“ (S. 1594)
Wong A, Trollope-Kumar K. Reflections: An inquiry into medical students‘ professional identity formation. Med Educ. 2014;48(5):489-501. DOI: 10.1111/medu.12382	„Professional identity formation can be conceptualised as the process by which an individual self-defines as a member of that profession based on the acquisition of the requisite knowledge, skills, attitudes, values and behaviours.“ (S. 490)
Yardley S, Kinston R, Lefroy J, Gay S, McKinley RK. ‚What do we do, doctor?‘ Transitions of identity and responsibility: A narrative analysis. Adv Health Sci Educ Theory Pract. 2020;25(4):825-43. DOI: 10.1007/s10459-020-09959-w	„We heard junior doctors recount their experiences of transition and the perceptible steps between accepting the mantle, the external imposition of responsibility and the requirement to act autonomously prior to the internal change in their sense of self. Successfully enacting this requirement creates identity capital (Côté 1997; Côté and Levine 2002) which accumulates and stimulates a metamorphosis when they can acknowledge their growth in self-confidence and proficiency. With this comes completion of the transition into the identity of being a doctor.“ (S. 840f)
