# Supplementary material for: Professional Identity Formation in medical education and training – a discursive determination of the term in German-speaking contexts
Source: GMS J Med Educ. 2026 Mar 23;43(3):Doc35. doi: 10.3205/zma001829 (PMC13054823; doi:10.3205/zma001829)
Supplement: Attachment 2: Phase 2 [file JME-43-35-s-002.pdf]

Attachment 2: Phase 2

Attachment 2 to Schick K, Kuehlmeyer K, Jömann B, Schumm M, Mathes S, Homberg A. *Professional Identity Formation in medical education and training – a discursive determination of the term in German-speaking contexts*. GMS J Med Educ. 2026;43(3):Doc35. DOI: 10.3205/zma001829

Attachment 2: Phase 2
a. Questionnaire for the asynchronous online survey

1. Professional identity development is an iterative process.

a. Do you agree with this statement?

1	2	3	4
1 = strongly disagree			4 = agree

b. Could you please explain your answer?

c. You have the option of formulating an alternative sentence here.

2. It is self-organized and externally organized.

a. Do you agree with this statement?

1	2	3	4
1 = strongly disagree			4 = agree

b. Could you please explain your answer?

c. You have the option of formulating an alternative sentence here.

3. This process takes place during a medical person´s confrontation with their self-image, their experiences and external (role) expectations that are placed on them.

a. Do you agree with this statement?

1	2	3	4
1 = strongly disagree			4 = agree

b. Could you please explain your answer?

c. You have the option of formulating an alternative sentence here.

4. In this process, (prospective) physicians acquire and internalize knowledge, skills, and values in the sense of a deeply rooted attitude, to be a good physician.

a. Do you agree with this statement?

1	2	3	4
1 = strongly disagree			4 = agree

b. Could you please explain your answer?

c. You have the option of formulating an alternative sentence here.

5. What being a good physician means must be contextualized in the respective social context.

a. Do you agree with this statement?

1	2	3	4
1 = strongly disagree			4 = agree

b. Could you please explain your answer?

c. You have the option of formulating an alternative sentence here.

6. Is there anything else you would like to add?

b. Results of the asynchronous online survey

Sentence	Md (IQR)	Reason for assessment	Alternative Formulation
Professional identity development is an iterative process.	4<br><br>(1,00)	- Different understanding of "iterative"<br><br>- The context is missing (target group and time period)	- ... is an ongoing/continuous/ conscious process.<br><br>- … is an iterative process that accompanies the entire medical professional life.<br><br>- … is a maturing process.<br><br>- … process in all phases of a physican's life from the beginning of their studies to the end of their career.<br><br>- … which is characterized by the studies and the years as a physician.<br><br>- … which encompasses all phases of life/the entire medical lifespan.
It is self-organized and externally organized.	3<br><br>(1,25)	- "it is" is too passive, incomprehensible<br><br>- "organized": questionable whether this is the case, socialization and identity development are also passive and unintentional.<br><br>- "self and externally" both aspects important, but the terms do not fit together, too unspecific	- It takes place actively.<br><br>- It is self- and externally controlled/ externally supported/ organized by others.<br><br>- It is driven/powered by external and internal factors/self-determined and externally determined factors.<br><br>- It is an active process of relating oneself in professional situations and contexts.<br><br>- It is highly self-directed.<br><br>- It takes place both by oneself and externally supported/organized by others.<br><br>… and can be supported through targeted learning and external experience opportunities.
This process takes place during a medical person´s confrontation with their self-image, their experiences and external (role) expectations that are placed on them.	3<br><br>(2,00)	- "medical person" sounds strange, does not include the students<br><br>- Describe more clearly what comes from inside and outside.<br><br>- Experiences can not be placed on them.<br><br>- The sentence is unwieldy, incomprehensible.	- … in the partly conscious, partly unconscious confrontation ...<br><br>- ... of the aspiring/becoming/ future physician/an individual/ of medical students or physicians<br><br>- … with external ideas and expectations regarding the medical role and behavior.<br><br>-… with the profession-related/ medical self-image/ own understanding of the role/ unconscious imprints and reactive attitudes<br><br>- ... in the interaction of external factors and their own understanding of their role ...<br><br>- ... in the intrinsically and extrinsically driven positioning in the interplay of personal freedom and professional responsibility.

Sentence	Md (IQR)	Reason for assessment	Alternative Formulation
			- In addition, the role expectations placed on her/him from outside are reflected upon and influence the further development process.
In this process, (prospective) physicians acquire and internalize knowledge, skills, and values in the sense of a deeply rooted attitude, to be a good physician.	3<br><br>(0,25)	- values cannot be acquired<br><br>- unclear whether PIF is an attitude or encompasses all areas of competence<br><br>- concept of attitude unclear<br><br>- “Deeply rooted” does not bring any added value	- … knowledge, skills and reflected (moral) values/attitude ...<br><br>- delete “to be a good physician”<br><br>- … attitude that they need to be a good physician/ provides a basis for becoming and being a good physician.<br><br>…be acquired and consolidated in the sense of a deeply rooted attitude.
What being a good physician means must be contextualized in the respective social context.	3,5<br><br>(1,00)	- is not value-free<br><br>- is too arbitrary<br><br>- unclear what is meant by it	- How a medical professional fills the role well ...<br><br>- What ideas, expectations and ideals are directed at (prospective) physicians ...<br><br>- Which norms, rights and duties are relevant to their work and which skills they need for the successful practice of their profession ...<br><br>- … needs to be concretized in the respective social context.<br><br>… needs to be placed in the context of existing social discourses and conventions.<br><br>… is determined by self-imposed demands on the medical profession and societal expectations of its practice.<br><br>… is the subject of an ongoing social, constitutional and ethical discourse.

Note: Response Scale: 1 = strongly disagree, 4 = agree; Md = Median, IQR = interquartile range; reasons for assessment and alternative wording were paraphrased; N = 16

c. Results of the second workshop

Group	Sentence	Suggested wording	Discussion points
A<br>N = 4	Professional identity development is an iterative process.	The professional identity development/ identity formation of physicians is an ongoing/continuous/iterative process in all phases of professional training and work.	- Usage of the term "formation" or "development"<br><br>- Alternatives to "persistent": iterative, cyclical, ongoing, continuous
	It is self-organized and externally organized.	It is subject to internal and external influences that can be addressed and shaped in medical education, further education and training.	- Sentence could be deleted if necessary<br><br>- Unclear whether designed or organized is the better term.
B<br>N = 4	This process takes place during a medical person's confrontation with their self-image, their experiences and (role) expectations that are placed on them.	The process is partly conscious, partly unconscious and partly reflective.<br>It is subject to internal and external influences, which can be taken up and shaped/organized in medical education, further education and training. It is formed in the interaction between the individual self-image of the aspiring/future physicians and their environment, which consists of expectations, values, norms and role models.	- Unclear whether "identity formation" or "development" is the better term (education ties in with the educational concept)<br><br>- Possible metaphor for the process: spiral or plateau as opposed to stages/progression<br><br>- Unclear whether "aspiring" or "future" is the better term
C<br>N = 4	In this process, (prospective) physicians acquire and internalize knowledge, skills, and values in the sense of a deeply rooted attitude, to be a good physician.	In this process, they acquire knowledge and skills in order to take responsibility for the medical tasks entrusted to them. Furthermore, they develop a self-reflective attitude in the context of the norms and values of their profession.	- The term "deeply rooted" doubles with "internalize"<br><br>- "Self-reflective" is a prerequisite for becoming a good physician<br><br>- It should be noted that students do not start the process as a blank canvas.
	What being a good physician mens must be contextualized in the respective social context.	These values and norms are constituted discursively.	- The term "good physician" is normative, unspecific, possibly not practicable<br><br>- "constituted discursively" is a sociological term

Note: The suggested wording and discussion points are taken from the small groups' presentation of their results.
