# Supplementary material for: Professional Identity Formation in medical education and training – a discursive determination of the term in German-speaking contexts
Source: GMS J Med Educ. 2026 Mar 23;43(3):Doc35. doi: 10.3205/zma001829 (PMC13054823; doi:10.3205/zma001829)
Supplement: Attachment 3: Phase 3 [file JME-43-35-s-003.pdf]

Attachment 3: Phase 3

Attachment 3: Phase 3
1.a. Questionnaire Group Delphi 1

Shared Understanding of „Professional Identity Formation“

A Group Delphi with members of the GMA working group „Professional Identity Formation“ to develop a shared understanding of PIF for medical education, further education, and continuing training in German-speaking countries

Dear Colleagues,

We are delighted that you are participating in this Group Delphi. The goal is to develop a shared understanding of "Professional Identity Formation".

This questionnaire was developed based on the following previous work:

At the working group meeting in January 2023, a preliminary draft was developed during a workshop based on selected relevant literature:

> *PIF is an iterative process. It is self-organized and externally organized. This process takes place during a medical person's confrontation with their self-image, their experiences and external (role) expectations that are placed on them. In this process, (prospective) physicians acquire and internalize knowledge, skills and values in the sense of a deeply rooted attitude, to be a good physician. What being a good physician means must be contextualized in the respective social context.*

This was commented on and further developed through an online survey (n = 16). At our working group meeting in January 2024, the description was dissected and discussed in groups. The following revised version resulted:

> *The professional identity development/ identity formation of physicians is a(n) ongoing/continuing/iterative process in all phases of professional training and work. The process is partly conscious, partly unconscious and partly reflective. It is subject to internal and external influences, which can be adopted and shaped/organized in medical education, further education, and continuing training. It is formed in the interaction between the individual self-image of the aspiring/ future physicians and their environment, consisting of expectations, values, norms and role models. In this process, they acquire knowledge and skills. Furthermore, they develop a self-reflective attitude in the context of the norms and values of their profession. These values and norms are constituted discursively.*

This questionnaire contains the proposals for phrasing developed at the working group meeting 2024, which are divided into the following areas:

A Subject of the definitionn
B Process of PIF
C Aims of PIF

Instructions for completing the questionnaire

Please complete the questionnaire in your small group. The individual items in sections A-C should be evaluated on a scale from **1 = no agreement to 8 = full agreement.** In the next session, the sentence components will be reduced - provided there is consensus and agreement.

A consensus on the wording is reached when there is a low dispersion of answers (IQR < 2). Only ratings on a scale level 5 and above are considered as agreement. Wordings with high dispersion or lack of agreement will be discussed in the plenary session of the next meeting and can be modified for the next round or deleted if there is a consensus about their rejection.

Please ensure that you do not spend more than 30 minutes on a section/page initially, and allocate the available time during this process.

**A Subject of the inition**									
Please indicate whether you do not agree at all (1) or completely agree (8) with the wording. Please select one value per line if possible		No agreement			Neither agree nor disagree				Full agreement
		**1**	**2**	**3**	**4**	**5**	**6**	**7**	**8**
**A0**	The professional identity development/ identity formation of physicians is a(n) ongoing/continuing/ iterative process in all phases of professional education and work.	○	○	○	○	○	○	○	○
**A1a**	The professional **identity development** of physicians ...	○	○	○	○	○	○	○	○
**A1b**	The professional **identity formation** of physicians ...	○	○	○	○	○	○	○	○
**A2a**	is an **ongoing** process ...	○	○	○	○	○	○	○	○
**A2b**	is a **continuing** process ...	○	○	○	○	○	○	○	○
**A2c**	is an **iterative** process ...	○	○	○	○	○	○	○	○
**A3**	in all phases of professional education and work.	○	○	○	○	○	○	○	○

B Process of PIF									
Please indicate whether you do not agree at all (1) or completely agree (8) with the wording. Please select one value per line if possible.		No agreement			Neither agree nor disagree				Full agreement
		**1**	**2**	**3**	**4**	**5**	**6**	**7**	**8**
**B1.0**	The process is partly conscious, partly unconscious and partly reflective. It is subject to internal and external influences, which can be adopted and shaped/organized in medical education, further education and training.	○	○	○	○	○	○	○	○
**B1.1**	The process is partly conscious, partly unconscious and partly reflective.	○	○	○	○	○	○	○	○
**B1.2**	It is subject to internal and external influences, ...	○	○	○	○	○	○	○	○
**B1.3 a**	which can be adopted and **shaped** in medical education, further education and training.	○	○	○	○	○	○	○	○
**B1.3 b**	which can be adopted and **organized** in medical education, further education and training.	○	○	○	○	○	○	○	○
**B2.0**	It is formed in the interaction between the individual self-image of the aspiring/ future physicians and their environment, consisting of expectations, values, norms and role models..	○	○	○	○	○	○	○	○
**B2.1**	It is formed in the interaction between the individual self-image...	○	○	○	○	○	○	○	○
**B2.2 a**	of the **aspiring** physicians ...	○	○	○	○	○	○	○	○
**B2.2 b**	of the **future** physicians ...	○	○	○	○	○	○	○	○
**B2.3**	and their environment, ...	○	○	○	○	○	○	○	○
**B2.4**	consisting of expectations, values, norms and role models.	○	○	○	○	○	○	○	○

C Aims of PIF									
Please indicate whether you do not agree at all (1) or completely agree (8) with the wording. Please select one value per line if possible.		No agreement			Neither agree nor disagree				Full agreement
		1	2	3	4	5	6	7	8
C0	In this process, they acquire knowledge and skills [in order to take responsibility for the medical tasks entrusted to them]. Furthermore, they develop a self-reflective attitude in the context of the norms and values of their profession. These values and norms are constructed discursively.	○	○	○	○	○	○	○	○
C1	In this process, they acquire knowledge and skills.	○	○	○	○	○	○	○	○
C2	... in order to take responsibility for the medical tasks entrusted to them.	○	○	○	○	○	○	○	○
C3	Furthermore, they develop a self-reflective attitude ...	○	○	○	○	○	○	○	○
C4	in the context of the norms and values of their profession.	○	○	○	○	○	○	○	○
C5	These values and norms are constructed discursively.	○	○	○	○	○	○	○	○

D Designation of the term		
	Please select a maximum of terms from the following list that you would include to the final wording from the Group Delphi.	Auswahl
**D1**	Definition	◯
**D2**	Working definition	◯
**D3**	Determination of the term	◯
**D4**	Understanding of the concept	◯
**D5**	Explanation of the meaning	◯

Feedback on the process

Thank you very much for your expertise and for taking the time to support us!

1.b. Questionnaire Group Delphi 2

Shared Understanding of „Professional Identity Formation“

2nd Round

A Group Delphi with members of the GMA working group „Professional Identity Formation“ to develop a shared understanding of PIF for medical education, further education, and continuing training in German-speaking countries

Dear Colleagues,
This is the revised questionnaire after the first round of the Group Delphi.

This questionnaire contains the proposed wording developed during the working group meeting, which is divided in the following areas:

A Subject of the definitionn
B Process of PIF
C Aims of PIF

The individual sentences of the definition developed and the individual terms that have not yet been agreed upon are evaluated.

Instructions for completing the questionnaire
Please complete the questionnaire in your small group. The individual items in sections A-C should be evaluated on a scale from **1 = no agreement to 8 = full agreement**. In the next session, the sentence components will be reduced - provided there is consensus and agreement.

A consensus on the wording is reached when there is a low dispersion of answers (IQR < 2). Only ratings on a scale level 5 and above are considered as agreement. Wordings with high dispersion or lack of agreement will be discussed in the plenary session of the next meeting and can be modified for the next round or deleted if there is a consensus about their rejection.

Please ensure that you do not spend more than 20 minutes on a section/page initially, and allocate the available time during this process.

A Subject of the definition									
Please indicate whether you do not agree at all (1) or completely agree (8) with the wording. Please select one value per line if possible.		No agreement			Neither agree nor disagree				Full agreement
		**1**	**2**	**3**	**4**	**5**	**6**	**7**	**8**
**A0**	The professional identity development/ identity formation of physicians is an ongoing/continuing/ iterative process in all phases of professional education and work.	◯	◯	◯	◯	◯	◯	◯	◯
**A1a**	The professional identity development of physicians is an ongoing process.	◯	◯	◯	◯	◯	◯	◯	◯
**A1b**	The professional identity development of physicians is an ongoing process **at all phases of professional education and work.**	◯	◯	◯	◯	◯	◯	◯	◯
**A1c**	The professional identity development of physicians is a **lifelong process.**	◯	◯	◯	◯	◯	◯	◯	◯

B Process of PIF									
Please indicate whether you do not agree at all (1) or completely agree (8) with the wording. Please select one value per line if possible.		No agreement			Neither agree nor disagree				Full agreement
		1	2	3	4	5	6	7	8
**B0**	The process is partly conscious, partly unconscious and partly reflective. It is subject to internal and external influences, which can be adopted and shaped/organized in medical education, further education and training.	○	○	○	○	○	○	○	○
**B1. 1a**	The process is **partly** conscious, **partly** unconscious.	○	○	○	○	○	○	○	○
**B1. 1b**	The process is **both** conscious and unconscious.	○	○	○	○	○	○	○	○
**B1.2**	It is subject to internal and external influences, which can be adopted and shaped in medical education, further education and training.	○	○	○	○	○	○	○	○
**B2.0**	It is formed in the interaction between the individual self-image of the aspiring/future physicians and their environment, which consists of expectations, values, norms and role models.	○	○	○	○	○	○	○	○
**B2. 1a**	**A professional identity develops** in the ...	○	○	○	○	○	○	○	○
**B2. 1b**	**A professional identity development involves** an ...	○	○	○	○	○	○	○	○
**B2. 1c**	A professional identity and the **self-image associated with it** develop in the …	○	○	○	○	○	○	○	○
**B2. 2a**	interaction of a person with their environment...	○	○	○	○	○	○	○	○
**B2. 2b**	interaction between person and environment.	○	○	○	○	○	○	○	○

C Aims of PIF									
Please indicate whether you do not agree at all (1) or completely agree (8) with the wording. Please select one value per line if possible.		No agreement			Neither agree nor disagree				Full agreement
		1	2	3	4	5	6	7	8
**C0**	In this process, they acquire knowledge and skills [in order to take responsibility for the medical tasks entrusted to them]. Furthermore, they develop a self-reflective attitude in the context of the norms and values of their profession. These values and norms are constructed discursively.								
**C1a**	In this process, individuals **develop** …	○	○	○	○	○	○	○	○
**C1b**	In this process, individuals **should develop** …	○	○	○	○	○	○	○	○
**C2**	… a (self-) reflective attitude ...	○	○	○	○	○	○	○	○
**C3a**	**… with regard to the ...**	○	○	○	○	○	○	○	○
**C3b**	**… in the context of ...**	○	○	○	○	○	○	○	○
**C4**	… norms and values of their profession (...).								

D Designation of the term		
	Please select all acceptable terms from the following list that we may use for the final formulation from the Group Delphi	Auswahl
**D1**	Definition	◯
**D2**	Working definition	◯
**D3**	Determination of the term	◯
**D4**	Understanding of the concept	◯
**D5**	Explanation of the meaning	◯

Thank you very much for your expertise and for taking the time to support us!

2. Quantitative results of the Group Delphi

Item		Item description	Round 1		Round 2	
Round 1	Round 2		Md (IQR)	Consensus	Md (IQR)	Consensus
**A**		**The professional identity development/ identity formation of physicians is a(n) ongoing/continuing/iterative process in all phases of professional education and work.**				
A1a)		The professional identity **development** of physicians ...	8 (0,50)			
A1b)		The professional identity formation of physicians ...	3 (1,25)			
A2a)	A1a	is an ongoing process ...	1,5 (2,00)			
A2b)		is an continuing process...	7,5 (1,50)		modified	
A2c)		.is an iterative process...	6 (2,00)			
	A1b)	The professional identity development of physicians is an ongoing process at all phases of professional education and work			6 (1,50)	
	A1c)	The professional identity development of physicians is a lifelong process.			5 (2,50)	
**B1**		**The process is partly conscious, partly unconscious and partly reflective. It is subject to internal and external influences, which can be adopted and shaped/organized in medical education, further education and training.**				
B1.1		The process is partly conscious, partly unconscious and partly reflective.	4,5 (1,50)		modified	
	B1.1a	The process is partly conscious, partly unconscious.			6 (3,00)	
	B1.1b	The process is both conscious and unconscious.			8 (2,50)	
B1.2	B1.2	It is subject to internal and external influences, ...	8 (1,25)			
B1.3a		which can be adopted and shaped in medical education, further education and training.	6,5 (1,25)			
B1.3b		which can be adopted and organized in medical education, further education and training.	1,5 (1,25)			
**B2**		**It is formed in the interaction between the individual self-image of the aspiring/ future physicians and their environment, consisting of expectations, values, norms and role models.**				
B2.1		It is formed in the interaction between the individual self-image...	6 (3,00)		modified	
B2.2a		of the aspiring physicians ...	3 (4,25)			
B2.2b		of the future physicians ...	3 (4,50)			
	B2.1a	A professional identity develops in the ...			7 (1,00)	
	B2.1b	A professional identity development involves an...			5 (1,50)	
	B2.1c	A professional identity and the self-image associated with it develop in the...			8 (2,50)	
B2.3		.and their environment, ...	8 (0,75)		modified	
	B2.2a	interaction of a person with their environment.			5 (1,00)	
	B2.2b	interaction between person and environment.			8 (0,50)	
B2.4		consisting of expectations, values, norms and role models.	4 (4,50)			

Item		Itembeschreibung	Runde 1		Runde 2	
Runde 1	Runde 2		Md (IQR)	Konsens	Md (IQR)	Konsens
**C**		**In this process, they acquire knowledge and skills [in order to take responsibility for the medical tasks entrusted to them]. Furthermore, they develop a self-reflective attitude in the context of the norms and values of their profession. These values and norms are constructed discursively.**				
C1		In this process, they acquire knowledge and skills...	4 (3,25)			
	C1a	iIn this process, individuals **develop** …			6 (3,00)	
	C1b	iIn this process, individuals **should develop …**			7 (2,50)	
C2		... in order to take responsibility for the medical tasks entrusted to them.	1 (0,00)			
C3		Furthermore, they develop a self-reflective attitude...	4 (2,25)			
	C2	…. a (self-) reflective attitude ...			8 (0,50)	
C4		in the context of the norms and values of their profession.	7,5 (1,00)			
	C3a	…. with regard to the norms and values of their profession.			6 (2,00)	
	C3b	… in the context of the norms and values of their profession.			8 (0,50)	
C5		These values and norms are constructed discursively.	4 (2,50)			

Note. Consensus: green and blue: Agreement (Md > 5, IQR < 2), blue: this has been deleted due to a higher-rated alternative being available; yellow: Dissent (Md = [4; 5] or IQR > 2); red: Rejection (Md < 4).

3. Qualitative results of the Group Delphi

Round	Sentence	Discussion points	Arguments for decision
3a	The professional identity **development/** identity **formation** of physicians is a(n) …	- formation (Bildung) or development (Entwicklung)	- “development” is a more “neutral” term<br>- “development” better expresses the processual nature of the concept<br>- “development” puts the subject at the center<br>- “development” can be shaped by the subject (work on oneself)<br>- “formation” appears externally influenceable, passive, finite and static<br>- “formation” is conceptually closely related to education
		- professional group of physicians	- focus on this professional group reduces complexity<br>- the focus does not exclude other professional groups
3b	**...ongoing/continuing/iterative** process...	- qualifying the process	- “continuing” emphasizes a development process until the end of life<br>- “continuing” implies a broad and open process design<br>- “ongoing” evokes associations of an illness<br>- “ongoing” offers no added value to the process term<br>- “iterative” is a less familiar foreign-word<br>- “iterative” evokes associations of a “monotonous” process<br>- “iterative” implies a mechanism of action (e.g. a repetition, a back-and-forth movement or that several sides would act reciprocally in relation to each other), which does not necessarily have to be the case<br>- “lifelong” refers to the importance of the private sphere and the phases before education and during retirement, but neglects the link to professional activity
3b	... along**/in all phases of professional education and work.**	- phases	- intuitively, phases are associated with medical education programs (professional education, further education, and continuing training)<br>- thus, it also indirectly narrows down the time when PIF is getting more important (at the beginning of professional education) and when it is getting less important (at the end of professional activity/retirement), whereas “along” here has a more restrictive effect than “in all phases”<br>- if the phases referred to PIF instead of professional education and work, alternative models, such as a continuum, would be more convincing.
		- restriction to the professional	- the addition is necessary to emphasize that PIF can be shaped trough education programs<br>- a strategic objective is associated with the definition of the term<br>- nevertheless, influences can also be located in the private (personal) sphere (e.g., in the family, in the reflection with mentors outside of working hours)

3b	It [the process] is formed in the interaction between the individual self-image of the aspiring/ future physicians and their environment, consisting of expectations, values, norms and role models.<br><br>**A professional identity develops in the / A professional identity development involves an.../**A professional identity and the **self-image associated with it** develop in the/interaction of a person with their environment/interaction between person and environment	- the genesis of a process or identity	- the phrase “is formed” sounds passive and self-evident, whereas „develops” seems more active, concrete, explicit and malleable<br>- it is a self-understanding that matures in development<br>- instead of referring to (aspiring/future) physicians, it is better to refer to “the person”<br>- “professional identity development involves” seems like a definition within a definition<br>- “involves” makes no claim to completeness, which makes the statement seem arbitrary
		- mentioning the professional self image	- the professional self-image is a part of PIF, but it does not make a decisive contribution to the clarification here
		- interaction between person and environment	- „between person and environment” is better because influences are possible in both directions
		- expectations, values, norms, and role models	- the necessity of establishing a connection between the environment and the narrow focus on “expectations, etc.” is questionable<br>- background information on the definition of “environment” should not be included in the determination of the term<br>- not everything listed here is also a component of the environment, for example internalized values and role models<br>- the discussion of other aspects, such as other professional groups, is also important but is missing
	.		
3c	The process is **partly** conscious, **partly** unconscious. / The process is **both** conscious and unconscious.	- relationship between the conscious and the unconscious parts	- “both” seems to be clearer, more specific, and more complete<br>- “both” describes a comprehensive process<br>- “both” indicates that the two aspects can be addressed equally, even though an “unconscious” processes cannot be influenced directly<br>- “partly, partly” seems unfinished, colloquial, and terse<br>- “partly, partly” denotes a simultaneous occurrence that does not necessarily have to happen<br>- “partly, partly” falsely implies that one knows how the single parts work

		- relationship between the conscious and the unconscious parts	- Both pairs of terms - "conscious/unconscious" and "reflected/unreflected" are too much<br>- "conscious" and "reflected" are not synonyms (statements can be both conscious and unreflected)<br>- "reflection" is important, but it is not always a given and needs to be supported
3a	**It is subject to internal and external influences, which can be adopted and shaped /organized in medical education, further education and training.**	- internal and external influences	- "Internal and external influences" are not required, as they are linked to "conscious/unconscious"<br>- "Internal and external influences" also refer to factors outside of education programs, such as the hidden curriculum
		- shaped in medical education, further education and training	- opening up a creative space for medical education while simultaneously restricting it<br>- PIF can also occur outside the scope of education programs<br>- "shape" seems more free, more open, and more active, whereas "organize" has a rather military connotation<br>- this could be a good starting point for designing the curriculum
3c	In this process, individuals **develop / should develop** knowledge and skills [in order to take responsibility for the medical tasks entrusted to them]. Furthermore, they develop/should develop a (self-)reflective attitude **in the context of/with regard to** the norms and values of their profession. These values and norms are constructed discursively.	- develop or should develop	- a self-reflective attitude is not guaranteed, which is why the educational aspiration formulated here is characterized by "should"<br>- PIF should be understood as an active process that can be influenced by teaching and mentoring services<br>- "should" implies that medical faculties are mandated to teach self-reflection skills<br>- "should" allows one to counteract the arbitrariness of a self-perpetuating development of PIF<br>- self-reflection is neutral and can be evaluated as either good or bad<br>- the main argument against "should" is that the phenomenon has existed for centuries without a specified direction; one cannot suddenly decide to design PIF with a normative goal<br>- on a meta-level: definitions should not be normative but descriptive, because this leaves more room for discussion and flexibility<br>- "is" in the end is at least true, since some kind of identity can be determined by the end of the study program
	These values and norms are constructed discursively.	- PIF and competency orientation	- PIF is a concept that can be understood in contrast to competency-oriented medicine<br>- PIF can also be understood as a concept that includes but goes beyond competency<br>- PIF is not contradicted by competency-based medical education<br>- at this point, the concept of competency is vague, which makes the statement unclear<br>- the term "competency" obscures the fact that PIF is primarily concerned with attitudes

		- PIF is also about "knowledge and skills," but these are situated in the context of values and norms<br>- the development of a “self-reflective attitude” is of central importance through the process of becoming aware of experiences
	- the context of professional norms and values	- professional norms and values are shaped by time and through society (e.g., the legalization of abortion has changed in the course of time)
	- exclusion of the origin of norms	- a statement about the origin of norms is unnecessary and goes beyond the determination of PIF<br>- in addition to the establishment of values and norms by society, there is also the establishment of norms by the medical profession<br>- professional norms are changeable, but in contrast core values are less negotiable<br>- a discursive constitution of norms involves an arbitrariness that can also leave the democratic value system
